# A cyclic-di-GMP receptor required for bacterial exopolysaccharide production

**DOI:** 10.1111/j.1365-2958.2007.05879.x

**Published:** 2007-09

**Authors:** Vincent T Lee, Jody M Matewish, Jennifer L Kessler, Mamoru Hyodo, Yoshihiro Hayakawa, Stephen Lory

**Affiliations:** 1 Department of Microbiology and Molecular Genetics, Harvard Medical School Boston, MA 02115, USA; 2 Department of Cell Biology and Molecular Genetics, University of Maryland College Park, MD 20742, USA; 3 Graduate School of Information Science/Human Informatics and CREST of JST, Nagoya University Chikusa, Nagoya 464-8601, Japan

## Abstract

Bis-(3′,5′)-cyclic-dimeric-guanosine monophosphate (c-di-GMP) has been shown to be a global regulatory molecule that modulates the reciprocal responses of bacteria to activate either virulence pathways or biofilm formation. The mechanism of c-di-GMP signal transduction, including recognition of c-di-GMP and subsequent phenotypic regulation, remain largely uncharacterized. The key components of these regulatory pathways are the various adaptor proteins (c-di-GMP receptors). There is compelling evidence suggesting that, in addition to PilZ domains, there are other unidentified c-di-GMP receptors. Here we show that the PelD protein of *Pseudomonas aeruginosa* is a novel c-di-GMP receptor that mediates c-di-GMP regulation of PEL polysaccharide biosynthesis. Analysis of PelD orthologues identified a number of conserved residues that are required for c-di-GMP binding as well as synthesis of the PEL polysaccharide. Secondary structure similarities of PelD to the inhibitory site of diguanylate cyclase suggest that a common fold can act as a platform to bind c-di-GMP. The combination of a c-di-GMP binding site with a variety of output signalling motifs within one protein domain provides an explanation for the specificity for different cellular responses to this regulatory dinucleotide.

## Introduction

Recently, bis-(3′,5′)-cyclic-dimeric-guanosine monophosphate (c-di-GMP) has been shown to be a central regulator in bacterial physiology that reciprocally regulates behaviour of bacterial communities (D'Argenio and Miller, 2004; Jenal, 2004; Romling *et al*., 2005; Romling and Amikam, 2006). In several bacterial species, a rise in c-di-GMP levels results in an increase in expression of various factors necessary for the establishment and maintenance of biofilm communities, whereas decrease in the production of the cyclic dinucleotide or its cleavage usually leads to enhanced expression of virulence and motility factors. In this signalling cascade, a number of controls exist to ensure proper responses to the levels of cellular c-di-GMP including signal generation, signal degradation and signal recognition. The level of c-di-GMP is regulated by the opposing activities of diguanylate cyclases (DGCs) that synthesize and phosphodiesterases (PDEs) that degrade this signalling molecule (Paul *et al*., 2004; Bobrov *et al*., 2005; Hickman *et al*., 2005; Schmidt *et al*., 2005; Christen *et al*., 2006; Kulasakara *et al*., 2006; Ryjenkov *et al*., 2006). These enzymes can be readily identified by their conserved GG(D/E)EF and EAL motifs respectively (Galperin *et al*., 2001) (Pfam Accession No. PF00990 and PF00563). Studies in a number of pathogenic bacteria have demonstrated the reciprocal relationship of DGC and PDE activities including those in *Vibrio cholerae* (Tamayo *et al*. 2005;Tischler and Camilli, 2005), *Salmonella typhimurium* (Simm *et al*., 2004; Romling, 2005; Simm *et al*., 2005;Kader *et al*. 2006), *Yersinia pestis* (Kirillina *et al*., 2004; Bobrov *et al*., 2005) and *Pseudomonas aeruginosa* (Hickman *et al*., 2005; Hoffman *et al*., 2005; Kulasakara *et al*., 2006).

The first biological process shown to be dependent on c-di-GMP was the production of cellulose in *Gluconacetobacter xylinus* (Ross *et al*., 1987). In this system, c-di-GMP acts as an allosteric activator for cellulose biosynthesis (Ross *et al*., 1990) by binding to the BcsA1 protein (Weinhouse *et al*., 1997), a component of the cellulose synthase complex. BcsA1 contains a conserved sequence that has been suggested to be the c-di-GMP receptor, the so-called PilZ domain (Amikam and Galperin, 2006). Recently, several proteins containing PilZ domains have been shown to bind c-di-GMP (Ryjenkov *et al*., 2006; Christen *et al*., 2007; Merighi *et al*., 2007; Pratt *et al*., 2007; Ramelot *et al*., 2007). However, it is likely that the number of different c-di-GMP-binding domains may be far greater than those with PilZ domains. There is evidence for the existence of protein domains other than PilZ that are capable of binding to c-di-GMP including the inhibitory site (I-site) of many DGCs (Garcia *et al*., 2004; Kirillina *et al*., 2004; Christen *et al*., 2006; Goymer *et al*., 2006; Malone *et al*., 2007) and all PDEs that bind and hydrolyse the dinucleotide (Bobrov *et al*., 2005; Schmidt *et al*., 2005; Christen *et al*., 2006; Kulasakara *et al*., 2006).

A systematic analysis of *P. aeruginosa* genes encoding DGCs and PDEs identified a phenotype that could not be linked to known proteins that contain a PilZ domain (Kulasakara *et al*., 2006; V.T. Lee, unpubl. data). In these studies, overexpression of DGCs led to a rise in the levels of c-di-GMP and the formation of multicellular communities (pellicles) at the air–surface interface of the bacterial culture (Kulasakara *et al*., 2006). Pellicle formation in *P. aeruginosa* requires proteins encoded in the *pel* and *psl* operons to synthesize glucose and mannose-rich polysaccharides, respectively (Friedman and Kolter, 2004a, b; Jackson *et al*., 2004; Matsukawa and Greenberg, 2004; Vasseur *et al*., 2005). Expression of the *pel* genes required for PEL polysaccharide production in *P. aeruginosa* can be induced by several mechanisms. One pathway occurs through histidine kinases RetS and LadS that act in opposing manner on the GacA/GacS two-component system. The GacA/GacS system in turn controls the transcription of two small regulatory RNAs (srRNAs) *rsmY* and *rsmZ* leading to a decrease or increase in the translation of the *pel* operon transcripts in the presence of the RetS or LadS signal respectively (Goodman *et al*., 2004; Ventre *et al*., 2006). In a second pathway, increasing the levels of c-di-GMP also results in the increased transcription of *pel* operon expression as demonstrated by either overexpression of DGC (Kulasakara *et al*., 2006) or a mutation in *wspF*, a gene encoding a methylesterase, that indirectly elevates the levels of c-di-GMP presumably by affecting the activity of WspR diguanylate cyclase (Hickman *et al*., 2005). Analysis of mRNA levels using microarrays showed that the levels of various *pel* gene transcripts increased by 3- to 32-fold in *P. aeruginosa*Δ*retS* as compared with the parental strain (Goodman *et al*., 2004) and deletion of *wspF* resulted in comparable effect on *pel* transcripts (Hickman *et al*., 2005). Therefore, expression of *pel* genes and formation of the Pel polysaccharide appears to be controlled by several transcriptional and post-transcriptional mechanisms. However, there are no known PilZ domain-containing proteins encoded by the *pel* operon suggesting that another yet to determined protein domain may interact directly with c-di-GMP.

Here we report that c-di-GMP directly affects the ability of *P. aeruginosa* to form biofilms by mediating the production of the extracellular PEL polysaccharide. We show that PelD, encoded by one of the genes within the *pel* operon, specifically binds c-di-GMP. The expression of PelD and its binding of c-di-GMP are required for PEL polysaccharide production. A systematic analysis of the residues important for c-di-GMP binding in PelD identified a unique binding sequence that was conserved among many PelD orthologues. Whereas PelD does not share any primary sequence or secondary structural similarities to the known c-di-GMP-binding PilZ domain, it appears to have the conserved RxxD motif found in the I-site of PleD (Chan *et al*., 2004; Christen *et al*., 2006; Malone *et al*., 2007). However, the c-di-GMP-binding motif in PelD is distinct from the I-site of DGCs which have been previously shown to reside six to nine amino acids N-terminal to the GGEEF motif of these enzymes (Christen *et al*., 2006). Furthermore, c-di-GMP binding alters these proteins in a distinct manner to activate PelD for PEL synthesis in contrast to inhibition of diguanylate cyclase activity of PleD. Identification of a novel family of c-di-GMP-binding proteins allows the understanding of the diverse biological effects of this dinucleotide.

## Results

### Role of c-di-GMP in biofilm formation is distinct from the RetS signalling pathway

**Fig. 1:**
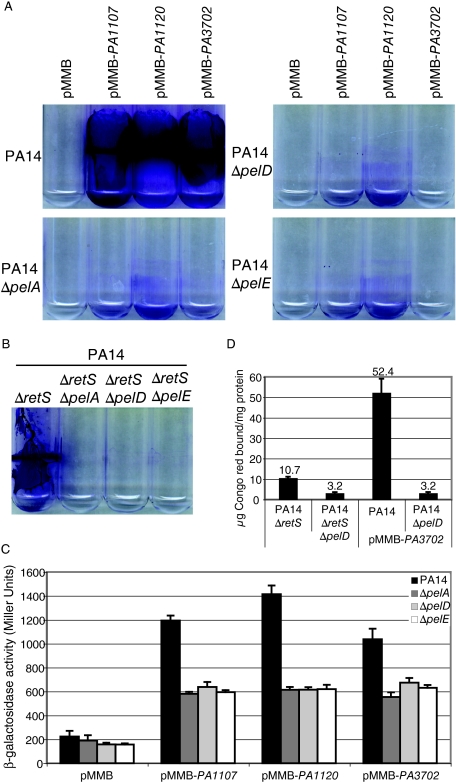
Phenotypic comparison of Δ*retS* mutation or overexpression of diguanylate cyclase on pellicle production and *pel* mRNA levels. A and B. Pellicle formation in static culture as revealed by crystal violet staining for (A) PA14, PA14Δ*pelA*, PA14Δ*pelD* and PA14Δ*pelE* harbouring vector control or induced for expression of diguanylate cyclases *PA1107*, *PA1120* or *PA3702*; (B) PA14Δ*retS*, PA14Δ*retS*Δ*pelA*, PA14Δ*retS*Δ*pelD* and PA14Δ*retS*Δ*pelE*. C. Expression of *pel* was determined by measuring β-galactosidase levels using a *pelA–lacZ* reporter construct in strains PA14, PA14Δ*pelA*, PA14Δ*pelD* and PA14Δ*pelE* harbouring vector control or induced for expression of diguanylate cyclases *PA1107*, *PA1120* or *PA3702*. D. Quantification of PEL polysaccharide by binding to Congo red was normalized to total protein for strains PA14 pMMB-*PA3702*, PA14Δ*pelD* pMMB-*PA3702*, PA14Δ*retS* and PA14Δ*retS*Δ*pelD*.

In-frame deletions of the *pelA*, *pelD* and *pelE* genes abrogated the ability of *P. aeruginosa* PA14 to produce biofilms in static culture in either the wild-type or Δ*retS* genetic background (Fig. 1A and B) (Vasseur *et al*., 2005). These results suggest that genes encoded by the *pel* operon are essential for pellicle formation. Using a *lacZ* transcriptional reporter fused to the *pel* promoter, we examined whether manipulating the levels of c-di-GMP by overexpressing proteins that have been previously shown to possess diguanylate cyclase activity would also effect the transcription of the *pel* operon (Kulasakara *et al*., 2006). In the wild-type PA14 parent carrying the pMMB vector alone, approximately 200 Miller units of β-galactosidase activity was detected and comparable expression of the *pel–lacZ* fusion was observed in the Δ*pelA*, Δ*pelD* or Δ*pelE* mutants (Fig. 1C). Induction of three independent diguanylate cyclases, PA1107, PA1120 and PA3702 (WspR), resulted in an increase in *pel* transcription in wild-type PA14 by five- to seven-fold, while in the Δ*pelA*, Δ*pelD* or Δ*pelE* backgrounds, transcription also increased by three-fold. These results indicate that high levels of cellular c-di-GMP enhance the level of *pel* mRNA. In each case, we consistently observed a higher induction of β-galactosidase in wild-type *P. aeruginosa* PA14 compared with Δ*pelA*, Δ*pelD* or Δ*pelE* mutants, suggesting a role for the functional PEL biosynthetic complex in regulating its own expression.

Biofilm formation by the Δ*retS* strain that accumulates elevated levels of *pel* transcripts and the strains overexpressing diguanylate cyclases appear to be qualitatively different with more biofilm material produced in the strains that are overexpressing diguanylate cyclase (Fig. 1A and B). We measured the amount of polysaccharide produced by each strain using a Congo red binding assay in which macromolecular polymers such as polysaccharides and protein filaments can sequester Congo red (Spiers *et al*., 2003). To account for the amount of bacteria in each biofilm, we normalized the Congo red binding to the amount of total protein in each sample. Biofilms, induced by overexpression of PA3702 (*wspR*), bound 52.4 ± 6.7 μg of Congo red per mg of protein; however, the biofilm produced by the Δ*retS* strain bound only 10.7 ± 0.6 μg of Congo red per mg of protein (Fig. 1D), a five-fold decrease as compared with DGC overexpression. *pelD* expression is required for pellicle production and polysaccharide production; Δ*pelD* pMMB-PA3702 and Δ*retS*Δ*pelD* strains bound only a minimal amount of Congo red (3 ± 2 μg of Congo red per mg of protein) (Fig. 1D). Using chemical analyses, we have separately demonstrated that Congo red binding to *P. aeruginosa* absolutely correlates with *pel*-dependent production of a hexose-containing polysaccharide (Table S1). These findings demonstrate that even when high level of *pel* operon transcript is present, it is the level of c-di-GMP which controls the amount of PEL polysaccharide produced by the bacteria and this signalling dinucleotide acts through the proteins encoded by the *pel* operon.

### Binding of c-di-GMP to proteins encoded by the *pel* operon

One possible mechanism for post-transcriptional regulation by c-di-GMP on increased pellicle production is by the direct interaction of c-di-GMP with a component of the PEL biosynthetic machinery encoded by the *pel* operon. A search for the previously identified c-di-GMP-binding PilZ domains among the *pel*-encoded proteins failed to identify any such domain. Therefore, a direct c-di-GMP binding assay was developed to assess the ability of recombinant Pel proteins to bind c-di-GMP.

**Fig. 2:**
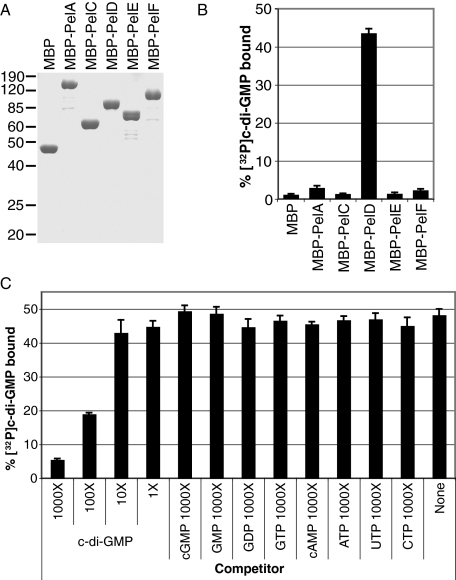
Identification of c-di-GMP-binding protein within the *pel* operon. A. Coomassie-stained SDS-PAGE of purified MBP fusions to PelA and domains of PelC, PelD, PelE and PelF lacking transmembrane segments. B. Binding of [^32^P]-c-di-GMP to each of the purified MBP-Pel fusion proteins. C. Specificity of c-di-GMP binding to MBP-PelD by competition with indicated unlabelled nucleotides.

Analysis of the proteins encoded by the *pel* operon indicates that PelA is a cytosolic protein, PelB is an outer membrane protein, PelC is a periplasmic protein (Lewenza *et al*., 2005), PelD and PelE are inner membrane proteins with large cytosolic domains, PelF shares homology to other glycosyltransferases and PelG is a 12-transmembrane inner membrane protein (Fig. S1). The *pel* operon is conserved in a number of bacterial species (Fig. S1). To determine whether any of the Pel proteins bind c-di-GMP, we inserted the *pel* genes in vectors that directed the synthesis of full-length PelA and PelF, and fragments of PelC, PelD and PelE lacking transmembrane domains fused to an N-terminal 6-histidine tag and maltose-binding protein (MBP). The purified proteins (Fig. 2A) were assayed for binding to [^32^P]-c-di-GMP by the ability of each protein bound to Ni-NTA agarose to retain radioactivity. Only the PelD fusion protein was able to retain [^32^P]-c-di-GMP (Fig. 2B). This interaction is specific as unlabelled c-di-GMP was able to block [^32^P]-c-di-GMP binding whereas other nucleotides including ATP, CTP, GTP, UTP, GDP, GMP, cyclic-GMP and cyclic-AMP did not compete for binding of the radiolabelled ligand (Fig. 2C). Furthermore, PelD does not bind [^3^H]-cyclic-GMP and [^3^H]-cyclic-AMP (Fig. S2). These results demonstrate that PelD specifically binds only c-di-GMP.

### Conserved residues in the C-terminal domain of PelD are required for binding of c-di-GMP

**Fig. 3:**
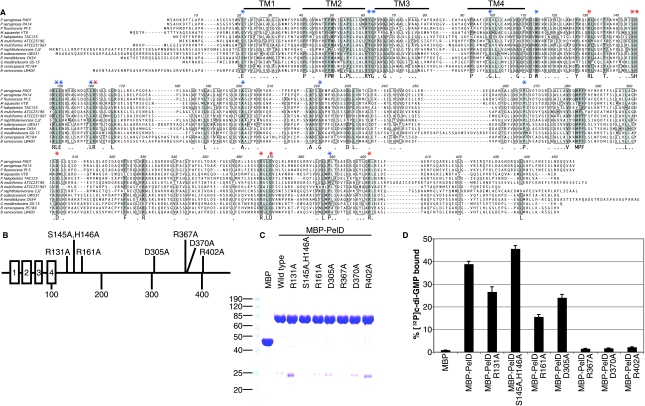
Role of conserved residues in PelD in c-di-GMP binding. A. Sequence alignment of PelD homologues. Similar residues are shaded in light grey and conserved residues (> 75%) are indicated by bold lettering. Residues conserved in all homologues are indicated by blue asterisks. Locations of point mutations introduced into PelD are indicate by red asterisks. B. Cartoon of the locations of introduced point mutations in the PelD sequence. C. Coomassie-stained SDS-PAGE of purified MBP-PelD with indicated point mutations. D. Binding of [^32^P]-c-di-GMP to each of the purified MBP-PelD fusion proteins.

Analysis of the PelD sequence using blast algorithms identified gene clusters in different bacterial species that encode PelD orthologues (Fig. 3A). Sequence alignment of PelD orthologues revealed conservation of the four transmembrane domains and an additional number of highly conserved residues (Fig. 3A). To avoid disrupting folding of the protein, we chose only hydrophilic and charged amino acids in the region C-terminal to the transmembrane segments as indicated in the schematic in Fig. 3B. The wild-type PelD and each of the point mutations were expressed as fusions to 6-histidine-MBP and were purified on Ni-NTA agarose (Fig. 3C). The purified proteins, immobilized on Ni-NTA agarose, were then tested for their ability to retain [^32^P]-c-di-GMP (Fig. 3D). PelD with substitutions R131A, R161A, D305A and a mutant with two substitutions, S145A/H146A, retained the ability to bind [^32^P]-c-di-GMP, whereas proteins containing R367A, D370A and R402A were unable to bind [^32^P]-c-di-GMP. These results identify three critical charged residues in the C-terminus of PelD that are required for the protein to bind c-di-GMP.

### Affinity of PelD for cyclic-di-GMP

**Fig. 4:**
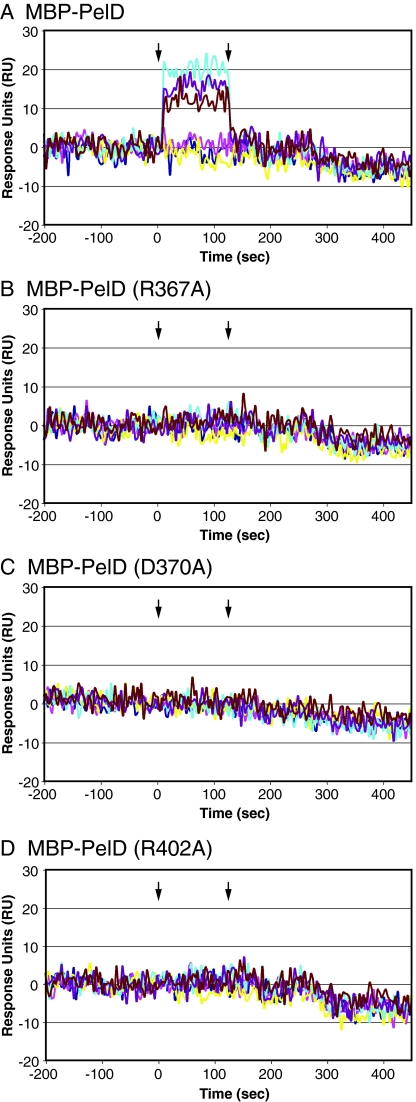
Affinity of PelD for c-di-GMP. MBP-PelD is chemically cross-linked on gold surface and probed with either c-di-GMP at 5 μM (brown), 10 μM (purple) or 20 μM (cyan) or GTP at 5 μM (yellow), 10 μM (pink) or 20 μM (blue) and washed away at times indicated by the arrows. A. MBP-PelD. B. MBP-PelD (R367A). C. MBP-PelD (D370A). D. MBP-PelD (R402A).

We determined the affinity of PelD for c-di-GMP using the surface plasmon resonance technique. The MBP-PelD or point mutant variants were chemically cross-linked onto gold surface. Either c-di-GMP or GTP at 5, 10 or 20 μM was passed over the protein-coated surface. An interaction between the protein and ligand results in a mass change of the complex that is detectable by a change in the resonance frequency of the gold surface and is read out as relative units (RU). MBP-PelD was able to bind c-di-GMP with a dissociation constant (*K*_D_) of 1 μM (Fig. 4A). Point mutants (R367A, D370A or R402A) were not able to bind c-di-GMP (Fig. 4B–D respectively). None of the MBP-PelD or point mutants bound GTP suggesting that PelD is not a diguanylate cyclase. The theoretical maximum response is proportional to the ratio of the molecular weight of the ligand and the protein and the number of binding sites for the ligand. As 6000 RU of protein was cross-linked onto each spot of the chip, the maximum response would be 50 RU for one c-di-GMP binding site and 100 RU for two c-di-GMP binding site. As the protein is chemically linked, it is very likely oriented in many directions relative to the surface and thus only a fraction, typically half, of the PelD would be available to bind c-di-GMP. The amount of response for MBP-PelD that approaches saturation is approximately 25 RU suggesting that there is one c-di-GMP binding site per PelD protein.

### PelD contains a cyclic-di-GMP-binding motif

**Fig. 5:**
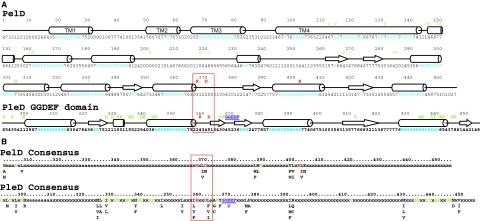
Secondary structure prediction and consensus sequence of PelD and PleD. A. Secondary structure predication was made using the web-based ProteinPredict program hosted at http://www.predictprotein.org/ for PelD and PleD. The confidence level of the prediction for each amino acid is located below the prediction. α-Helix and β-sheet elements predicted with a confidence level of greater than 5 is depicted on the diagram. Conserved residues for PelD and PleD are shown above the diagram and residues required for binding are shown in red. RxxD motif is indicated by the red box and GGEEF motif is in purple and is underlined. B. Consensus sequence of PelD and PleD was based on Phyre alignment of 20 and 100 homologues respectively. Residues required for c-di-GMP binding is indicated in red, GGEEF motif is in purple and is underlined and other conserved residues are in green. RxxD motif is indicated by the red box.

A Pfam search demonstrated that PelD lacked a recognizable PilZ domain in contrast to the eight other *P. aeruginosa* genes that encode PilZ domains. Secondary structure of PelD predicted by ProteinPredict (Rost *et al*., 2004) suggest that the PelD protein consists of alternating α-helix–β-sheet–α-helix–β-sheet–α-helix that is distinct from the β-barrel fold of PilZ domains (Fig. 5A). Another known c-di-GMP-binding domain is the I-site of diguanylate cyclases that controls feedback inhibition as demonstrated for *Caulobacter crescentus* PleD (Chan *et al*., 2004). The I-site consists of an RxxD motif that is invariably located nine and six amino acids from the GGEEF (Christen *et al*., 2006). Despite a low level of sequence similarity between PelD and PleD, we sought to determine whether there are any secondary structural similarities between these two proteins. Secondary structure predicted by ProteinPredict produced results that matched the known structure determined by crystallography (Fig. 5) (Chan *et al*., 2004). An important component of this computational analysis is the confidence level assigned for each of the predicted elements ranging from 0 to 9 with 9 being the greatest confidence of the prediction. Using alignment tools at Phyre web server (http://www.sbg.bio.ic.ac.uk/phyre) (Kelley *et al*., 2000), the conserved residues in PleD for the top 100 homologues yielded a number of residues including the catalytic GGEEF motif and the RxxD I-site and allow the identification of conserved identical and similar residues (Fig. 5B). Mapping these residues on the crystal structure of the PleD revealed that the GGEEF motif and all other conserved residues map to one face of the diguanylate cyclase domain, whereas the RxxD motif is on the other face (Fig. S3). Comparison of the PelD and PleD secondary structures suggests that they have similar domain fold consisting of alternating α-helix–β-sheet–α-helix–β-sheet–α-helix (Fig. 5B). Furthermore, the RxxD residues required for binding c-di-GMP are present in both proteins at similar position within the domain (Fig. 5B). Despite these similarities, PelD is obviously lacking the GG(D/E)F motif of diguanylate cyclases as well as other conserved residues. Furthermore, the equivalent of PelD R402 is much less conserved in PleD-like homologues. Together these results suggest the potential of a wide array of proteins that can utilize a simple RxxD c-di-GMP-binding motif to regulate the activity of other portions of the protein.

### PelD binding to c-di-GMP is required for pellicle production

**Fig. 6:**
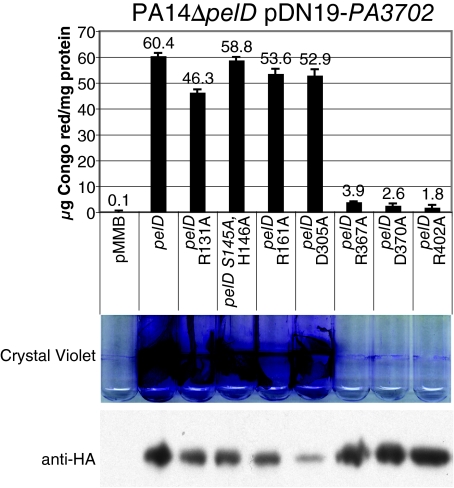
Residues required for c-di-GMP binding are required for PEL polysaccharide synthesis. Complementation of the Δ*pelD* mutation in PA14Δ*pelD* pDN19-*PA3702* with pMMB containing full-length *pelD* and indicated point mutations. Quantification of PEL polysaccharide binding to Congo red was normalized to total protein for each strain. Pellicle formation for each stain was tested in static culture and revealed by crystal violet staining. PelD and mutant derivatives were fused at their C-termini to HA tags. Total-cell extracts were prepared, loaded equally and separated on SDS-PAGE, transferred to PVDF and PelD-HA was detected with anti-HA antibody and chemiluminescence.

As *pelD* is required for pellicle production (Fig. 1A and B), we sought to determine whether binding of c-di-GMP to PelD is required for pellicle formation. A two-plasmid system was used to constitutively express a diguanylate cyclase (pDN19-PA3702), while *pelD* was induced from a second plasmid (pMMB). PA14Δ*pelD* harbouring pDN19-PA3702 was transformed with pMMB-Gn containing wild-type *pelD* and mutated versions of *pelD* genes, expressing PelD as a fusion protein with a C-terminal haemaglutinin (HA) tag. Expression of PelD mutants with R131A, R161A, D305A or S145A/H146A substitutions, which were unaffected in their ability to bind c-di-GMP, complemented the PA14Δ*pelD* mutant and restored its ability to form a robust pellicle and bind Congo red (Fig. 6). In contrast, PelD point mutants that are unable to bind c-di-GMP (R367A, D370A and R402A) failed to restore pellicle formation and did not bind Congo red. The observed phenotype of the PelD mutants R367A, D370A and R402A was not due to lower production of the mutated forms of these proteins, as they are detectable with an antibody to the HA tag (Fig. 5). These results demonstrate a correlation between the ability of PelD to bind c-di-GMP and its activity *in vivo* to participate in pellicle formation.

## Discussion

The cellular level of the second messenger c-di-GMP is controlled by the reciprocal activities of diguanylate cyclases and phosphodiesterases, which in many organisms are encoded by a large number of paralogous genes. One of the greatest challenges in the field is to understand the specificity and redundancy in the c-di-GMP regulatory pathway. Are there specific DGCs/PDEs responsible for activating or inhibiting specific pathways such as PEL production? If so, the DGC and PDE proteins may colocalize with the c-di-GMP receptors to allow for even greater sensitivity to signal generation by enhancing the effects of local concentration. Results from overexpression of DGCs have suggested that high levels of c-di-GMP results in functional redundancy as there is sufficient amount of c-di-GMP to occupy all cellular receptors. To establish the exact relationship of individual DGCs and PDEs with specific c-di-GMP mediated phenotypes will require the identification of all c-di-GMP protein in an individual organism.

Currently, only proteins with the PilZ domain have been shown to bind c-di-GMP other than enzymes involved in the metabolism of the dinucleotide. On metabolic enzymes that produce and degrade c-di-GMP, there are two sites that bind c-di-GMP including the putative regulatory domain of DGCs (Chan *et al*., 2004; Christen *et al*., 2006) and the substrate-binding domains of PDEs. In this article we presented the identification of a c-di-GMP-binding protein in *P. aeruginosa* that controls biofilm formation through the synthesis of an extracellular polysaccharide encoded by the *pel* operon. We purified each of the soluble fragments encoded by genes in the *pel* operon and found that only one component, PelD, specifically binds c-di-GMP. As the *pel* operon is conserved in a number of bacterial species, we aligned all PelD orthologues, identified conserved amino acids and engineered mutations in the *P. aeruginosa* PelD in codons for a number of conserved amino acids. Three mutations in the C-terminus of PelD resulted in the loss of c-di-GMP binding. When these three mutant PelD proteins were expressed in a PA14Δ*pelD* mutant background, these strains were unable to form biofilms and did not produce the PEL polysaccharide. These results demonstrate that binding of c-di-GMP to PelD is required for PEL polysaccharide synthesis. Additional production of c-di-GMP can increase binding to PelD and further activate PEL polysaccharide synthesis, possibly in a mechanism similar to the allosteric regulation of BcsA1 in activation of cellulose synthase in *G. xylinus* (Weinhouse *et al*., 1997). Findings from studies on PEL and alginate synthesis indicate that each polysaccharide biosynthetic machinery requires binding to c-di-GMP for activity and this mechanism of regulation may be a general theme for all polysaccharide biosynthetic systems regulated by c-di-GMP.

We have identified a c-di-GMP-binding protein that mediates the signal transduction of c-di-GMP towards expression of a surface polysaccharide. Bioinformatics analysis of the PelD protein sequence reveals that the protein secondary structure is comprised of alternating α-helices and β-sheets, which is in marked contrast to the PilZ domain that is comprised of four to five consecutive β-sheets to form a β-barrel (Ramelot *et al*., 2007). Other than PelD homologues in a limited number of bacterial species, database searches have failed to identify additional genes that encode proteins with the c-di-GMP-binding domain of PelD. Previously, Christen *et al*. (2006) have characterized in *C. crescentus* PleD DGC the regulatory I-site, containing the RxxD sequence, as a binding site for c-di-GMP and it is located six and nine amino acids from the GGEEF motif involved in synthesis of c-di-GMP. Although PelD and PleD are not related by primary sequence, they both contain the conserved RxxD residues. The similarities between the secondary structure of c-di-GMP-binding portion of PelD and the I-site of PleD suggest certain degree of structural conservation. Inspection of the structure of PleD for the location of the I-site revealed that it is found in a small binding pocket, which could function as the site for allosteric inhibition by c-di-GMP. The c-di-GMP-binding motif of PelD may be contained in such a similar pocket, but confirmation of this awaits structural determination of PelD. Nonetheless, the differences in the remaining portion of the two proteins, such as the lack of GGEEF motif in PelD, suggest that c-di-GMP-binding proteins may be present in many different proteins that lack sequence similarity to known c-di-GMP receptors. Furthermore, the flexibility in the sequence of c-di-GMP-binding domains allows the potential of both positive and negative allosteric regulation on a diverse set of proteins. This diversity of receptors for c-di-GMP may provide a mechanism for generation of specific responses following local production or degradation of this second messenger by the activities of multiple DGCs and PDEs encoded in bacterial genomes. As c-di-GMP binding is a requirement for the activation of a number of polysaccharides required for biofilm formation, inhibition of c-di-GMP production or interaction with its intracellular receptors presents attractive targets for the development of antimicrobial therapeutic agents against biofilm-related diseases.

## Experimental procedures

### Strains, plasmids and media conditions

Strains, plasmids and primers used in this study are listed in Tables S2, S3 and S4 respectively (Rahme *et al*., 1995). In-frame deletion of *retS*, *pelA*, *pelD* and *pelE* in PA14 was generated using the SOE strategy (Warrens *et al*., 1997). In short, 1 kb flanks of each gene were amplified by polymerase chain reaction (PCR) with internal primers containing overlapping sequences. Purified fragments from the first PCR and external primers were then used for a second round of PCR and the 2 kb product was cloned into pEX-Gn for integration into PA14. The cointegrant is resolved by counterselection on Luria–Burtani (LB) agar containing 6% sucrose and the deletion was verified by PCR using the external primers. *P. aeruginosa* strains were grown in LB broth. Plasmids pMMB-Gn containing *PA1107*, *PA1120* and *PA3702* (Furste *et al*., 1986; Kulasakara *et al*., 2006) as well as *pelD* and isogenic point mutants were maintained with 15 μg ml^−1^ gentamicin and expression was induced with 1 mM isopropylthiogalactose (IPTG). Plasmids pDN19 and pDN19-*PA3702* are described elsewhere (Nunn and Lory, 1991; J.M. Matewish, unpubl. data) and maintained with 15 μg ml^−1^ tetracycline. All amplified DNA were verified by sequencing.

### Crystal violet pellicle assay

Each strain was grown in LB containing appropriate antibiotics and IPTG as indicated in the text and incubated as a static culture at 37°C for 24 h. The culture medium was removed, the pellicle was washed with water and stained with 50 μg ml^−1^ crystal violet for 10 min, followed by the removal of unbound crystal violet by washing 10 times with distilled water.

### β-Galactosidase reporter assay

The promoter of the *pel* operon was cloned using primers listed in Table S4 and cloned in front of *lacZ* gene in pCTX–*lacZ*. The plasmid was integrated into the *ctx* site in the PA14 genome using the strategy described (Hoang *et al*., 1998). *P. aeruginosa* strains containing the *lacZ* reporter were grown in LB containing appropriate antibiotics and IPTG as indicated in the text. β-Galactosidase activity is measured according to an established method (Miller, 1992).

### Congo red binding assay

The assay was adapted from Spiers *et al*. (2003). Each strain was incubated in 1 ml of LB containing appropriate antibiotics and IPTG as indicated in the text for 24 h at 37°C in 2 ml microfuge tubes. The bacterial content was collected by centrifugation, re-suspended in 1 ml of 40 μg ml^−1^ Congo red in 1% tryptone and incubated for 90 min at 37°C at 250 r.p.m. The bacteria and bound Congo red were collected by centrifugation and the amount of Congo red remaining in the supernatant was determined by measuring the absorbance of the supernatant at 490 nm and compared with Congo red standard solutions.

### Protein expression and purification

Coding sequences of full-length PelA, PelC lacking its signal sequence (amino acids 16–172), the cytosolic fragments of PelD (amino acids 105–455), PelE (amino acids 95–329) and full-length PelF were amplified using the indicated primers by PCR, excised with appropriate restriction enzymes and cloned into pVL847 behind the 6-histidine-MBP tag. *pelD* point mutations were generated by QuikChange (Strategene, San Diego) using the following primer pairs described in Table S3. pVL847 and derivative plasmids were transformed into BL21. Expression of MBP and fusion proteins were induced by IPTG, purified over Ni-NTA agarose (Qiagen, California) and eluted by 250 mM imidazole. The imidazole is removed by PD10 desalting column (GE Healthcare, New Jersey) equilibrated with buffer containing 10 mM Tris, pH 7.4 and 100 mM NaCl. Proteins were aliquoted and frozen in liquid nitrogen.

### [^32^P]-c-di-GMP binding assay

[^32^P]-c-di-GMP is generated from [α-^32^P]-GTP using purified WspR. Each purified protein (1 mg ml^−1^) was incubated with 100 nM of [^32^P]-c-di-GMP for 30 min at 20°C with 100 μl of a 50% slurry of Ni-NTA agarose beads in 10 mM Tris, pH 7.4 and 100 mM NaCl. For competition experiments, various concentration of each indicated inhibitor is added after incubation of protein with [^32^P]-c-di-GMP and incubated for an additional 30 min. The beads with bound protein were collected by centrifugation and the supernatant was transferred to an empty microfuge tube for unbound [^32^P]-c-di-GMP. The beads were washed twice with 50 μl of 10 mM Tris, pH 7.4 and 100 mM NaCl and each wash was transferred to the same unbound [^32^P]-c-di-GMP tube. Both fractions were counted in the scintillation counter with a total of 500 000 counts in each sample. The percentage [^32^P]-c-di-GMP bound is calculated as 100 × the counts retained with the agarose beads divided by the sum of all counts.

### Surface plasmon resonance

All experiments were performed using a Bio-Rad ProteoOn XPR36 Protein Interaction array system. Each purified protein (1 mg ml^−1^) was cross-linked onto the surface of GLM chip using sulfo-NHS. The reaction was quenched with glycine and unbound protein is removed by washing. The amount of cross-linked protein was determined by change in molecular weight of the surface as RU. Solution of c-di-GMP or GTP at 5, 10 and 20 µM was passed over each spot of the chip and the change in molecular weight was detected.

## Supplementary material

The following supplementary material is available for this article:

Fig. S1 Operon organization of *pelA-G*. Predicted function, cellular location and alignment to homologous operons. Abbreviations under the genes are: SS, signal sequence; OM, outer membrane; PP, periplasmic; TM, transmembrane helix; IM, inner membrane. BLAST score for each *pel* gene to each homologue is indicated below each gene.

Fig. S2 PelD does not bind cyclic-GMP or cyclic-AMP. The ability of either MBP or MBP-PelD to bind ^3^H-c-di-GMP, ^3^H-cyclic-GMP or ^3^H-cyclic-AMP was assessed by using the nucleotide binding assay as described. Percent bound of each above background is shown.

Fig. S3 Location of I-site and GGDEF motif on opposing faces of the PleD. Location of RxxD (red), GGEEF (yellow) and other conserved residues (green) on PleD crystal structure. 1. Side view. 2. GGDEF face of PleD. 3. I-site face of PleD.

Table. S1 Correlation of hexose production and Congo red binding in *P. aeruginosa* PA14.

Table. S2 Strains used in this study.

Table. S3 Plasmids used in this study.

Table. S4 Primers used in this study.

This material is available as part of the online article from: http://www.blackwell-synergy.com/doi/abs/10.1111/j.1365-2958.2007.05879.x (This link will take you to the article abstract).
