# Supplementary material for: A cyclic-di-GMP receptor required for bacterial exopolysaccharide production
Source: Mol Microbiol. 2007 Sep;65(6):1474–84. doi: 10.1111/j.1365-2958.2007.05879.x (PMC2170427; doi:10.1111/j.1365-2958.2007.05879.x)

SUPPLEMENTAL INFORMATION

SUPPLEMENTAL TABLE I

Correlation of hexose production and congo-red binding in *P. aeruginosa* PA14.

		PA14 pMMB-PA3702	PA14Δ*pelD* pMMB-PA3702
Untreated biofilm	Congo red bound (ug)[b]	19.5 ± 2.0	0.9 ± 0.4
	Protein (μg)	382 ± 23	279 ± 8
Proteinase K treated[a]	Congo red bound (ug)[b]	13.7 ± 1.1	0.0 ± 0.0
	Protein (μg)	<50	<50
	Hexose content (ug)[a]	17.2 ± 0.4	0.0 ± 0.0

[a]Biofilms were incubated for 3 hours with 25 μg of proteinase K and 0.1% SDS.

[b]Congo red and hexose content are measured from insoluble material that is pelletable at 18,000 x *g* after indicated treatments.

Carbohydrate assay

The cells to be assayed were grown as biofilms in microfuge tubes as described above. Bacterial pellets were collected by centrifugation and resuspended in 0.1% SDS, 10 mM Tris-HCl (pH 8) and boiled for 1 min. The cell extract was cooled to room temperature, proteinase K was added to a final concentration of 50 ug/ml and incubated at 60°C for 3 hours. These preparations were centrifuged at 18,000 x *g* for 10 min and the pelleted carbohydrate material was washed once in water. The amount of carbohydrate material was determined by the phenol-sulfuric acid method (Dubois et al., 1956) with glucose as the standard. The protein content of the cell extracts before addition of proteinase K was determined using the Bradford protein assay.

SUPPLEMENTAL TABLE II – Strains

Strains	Relevant genotype	Reference and source
PA14	Wild type	Rahme *et al* (1995)
PA14Δ*pelA*	In-frame deletion of *pelA*	This study
PA14Δ*pelD*	In-frame deletion of *pelD*	This study
PA14Δ*pelE*	In-frame deletion of *pelE*	This study
PA14Δ*retS*	In-frame deletion of *retS*	Goodman *et al* (2004)
PA14Δ*retS*Δ*pelA*	In-frame deletion of *retS* and *pelA*	This study
PA14Δ*retS*Δ*pelD*	In-frame deletion of *retS* and *pelA*	This study
PA14Δ*retS*Δ*pelE*	In-frame deletion of *retS* and *pelA*	This study

SUPPLEMENTAL TABLE III – Plasmids

Plasmid	Relevant characteristic	Reference
pMMB	Low copy vector with *tac* promoter	Furste *et al* (1986)
pMMB-*PA1107*	pMMB with PA1107	Kulesakara *et al* (2005)
pMMB-*PA1120*	pMMB with PA1120	Kulesakara *et al* (2005)
pMMB-*PA3702*	pMMB with PA3702	Kulesakara *et al* (2005)
pDN19	Low copy vector with *tac* promoter	Nunn and Lory (1991)
pDN19-*PA3702*	pDN19 with PA3702	This study
pEX-Δ*pelA*	pEX with 1kb region upstream and 1kb downstream of *pelA*	This study
pEX-Δ*pelD*	pEX with 1kb region upstream and 1kb downstream of *pelD*	This study
pEX-Δ*pelE*	pEX with 1kb region upstream and 1kb downstream of *pelE*	This study
pCTX-pro-*pel*	pCTX 500bp upstream of *pelA*	This study
pVL847	pET19 with *malE*	This study
pVL876	pET19 with *malE* -*pelA* (full length)	This study
pVL874	pET19 with *malE* -*pelC* (amino acid 16-172)	This study
pVL880	pET19 with *malE* -*pelD* (amino acid 105-454)	This study
pVL873	pET19 with *malE* -*pelE* (amino acid 95-329)	This study
pVL871	pET19 with *malE* -*pelF* (full length)	This study
pVL1056	pET19 with *malE* -*pelD* (amino acid 105-454)	This study
pVL1057	pVL1056 with R131A mutation	This study
pVL1058	pVL1056 with SH145-6AA mutations	This study
pVL1059	pVL1056 with R161A mutation	This study
pVL1060	pVL1056 with D305A mutation	This study
pVL1061	pVL1056 with R367A mutation	This study
pVL1062	pVL1056 with D370A mutation	This study
pVL1063	pVL1056 with R402A mutation	This study
pVL1064	pMMB with *pelD*	This study
pVL1065	pMMB with *pelD* with R131A mutation	This study
pVL1066	pMMB with *pelD* with SH145-6AA mutations	This study
pVL1067	pMMB with *pelD* R161A mutation	This study
pVL1068	pMMB with *pelD* D305A mutation	This study
pVL1069	pMMB with *pelD* R367A mutation	This study
pVL1070	pMMB with *pelD* D370A mutation	This study
pVL1071	pMMB with *pelD* R402A mutation	This study
pVL1192	pMMB with *pelD-HA*	This study
pVL1193	pMMB with *pelD-HA* with R131A mutation	This study
pVL1194	pMMB with *pelD-HA* with SH145-6AA mutations	This study
pVL1195	pMMB with *pelD-HA* R161A mutation	This study
pVL1196	pMMB with *pelD-HA* D305A mutation	This study
pVL1197	pMMB with *pelD-HA* R367A mutation	This study
pVL1198	pMMB with *pelD-HA* D370A mutation	This study
pVL1199	pMMB with *pelD-HA* R402A mutation	This study

SUPPLEMENTAL TABLE IV – Primers		
Oligo-nucleotide name	Oligonucleotide sequence	Purpose
vl392	5'-AGAATTCAAAATCGAAGAAGGTAAACTGGTA	*malE* 5'-EcoRI
vl393	5'-AGCTAGCAGTCTGCGCGTCTTTCAGGGCT	*malE* 3'-NheI
vl367	5'-ACATATGATGCGGTTCAGCAAGAAAGGAATC	*pelA* 5'-NdeI
vl369	5'-ACCTCGAGTCAGCGGCAGACGAGTTGGCCAT	*pelA* 3'-XhoI
vl365	5'-ACATATGATGGCGGGCTGCTCCAGCTTCAC	*pelC* 5'-NdeI
vl366	5'-AGGATCCTCGCCGTCACTCGAGCCGCAGGTC	*pelC* 3'-BamHI
vl363	5'-ACATATGATGTTGGTGGGCGAGTTCCGCGACAT	*pelD* 5'-NdeI
vl370	5'-ACCTCGAGGAATGATCAGCAAGTGGCTGTTTAG	*pelD* 3'-XhoI
vl700	5'-AAGATCTAACAGCCACTTGCTGATCATTCAG	*pelD* 3'-BglII
vl362	5'-ACATATGCGCCAGCGTGGCGAGCAGGCCT	*pelE* 5'-NdeI
vl356	5'-ACATATGGATCCCGGTCATGTCCAGTATCTCGCCA	*pelE* 3'-BamHI
vl351	5'-ACATATGGAGATACTGGACATGACCGAACAC	*pelF* 5'-NdeI
vl352	5'-ACATATGCTCGAGCATGTCATGCAATCTCCGTGGCTT	*pelF* 3'-XhoI
vl577	5'-CTGGCGAACGAGTACCGCCAGCTAGCCCTCGACGAATTCACCCGCG	*pelD* R131A
vl578	5'-CGCGGGTGAATTCGTCGAGGGCTAGCTGGCGGTACTCGTTCGCCAG	*pelD* R131A
vl579	5'-CCATCACATCCTGCGCATCGCGGCCGATCGCCTGGAGCAGC	*pelD* S145A, H146A
vl580	5'-GCTGCTCCAGGCGATCGGCCGCGATGCGCAGGATGTGATGG	*pelD* S145A, H146A
vl581	5'-GCAACGACCAGAGCCTGGCGAGCTCGCTGCTCGGTCTGCGA	*pelD* R161A
vl582	5'-TCGCAGACCGAGCAGCGAGCTCGCCAGGCTCTGGTCGTTGC	*pelD* R161A
vl583	5'-TCGCCGGGCACATCGCCGCCTTGCTGCAGAGCGACCGT	*pelD* D305A
vl584	5'-ACGGTCGCTCTGCAGCAAGGCGGCGATGTGCCCGGCGA	*pelD* D305A
vl585	5'-CTGCTGGAAGGCAGCCAGGCAGGCCTCGACGTGCAGTTG	*pelD* R367A
vl586	5'-CAACTGCACGTCGAGGCCTGCCTGGCTGCCTTCCAGCAG	*pelD* R367A
vl587	5'-CAGCCAGCGTGGCCTCGCTGTACAGTTGCGACTGCGCAACGA	*pelD* D370A
vl588	5'-TCGTTGCGCAGTCGCAACTGTACAGCGAGGCCACGCTGGCTG	*pelD* D370A
vl589	5'-AGGCTCCCAGGGGTATCTGCAAGCTTTGAGGATTCTCTTCGCCGAA	*pelD* R402A
vl590	5'-TTCGGCGAAGAGAATCCTCAAAGCTTGCAGATACCCCTGGGAGCCT	*pelD* R402A
vl733	5'-AGTTAACCGGCACCAGCCACCTGTA	Δ*pelA* upstream 5'
vl734	5'-AGGATCCAGAGGAGCATATGTCTTTCTTGCTGAACCGCAT	Δ*pelA* upstream 3'
vl735	5'-ACATATGCTCCTCTGGATCCTTTGCCGATGGAGCAGGTG	Δ*pelA* downstream 5'
vl736	5'-AAAGCTTGCCAAGGCGCGCGAGAT	Δ*pelA* downstream 3'
vl491	5'-AGTTAACGGAGTGGGCCCACACACTGTTCT	Δ*pelD* upstream 5'
vl527	5'-AGGATCCAGAGGAGCATATGTCTTGTGCGCGGACATCGCCGTCA	Δ*pelD* upstream 3'
vl528	5'-ACATATGCTCCTCTGGATCCTAACGATCGCCAGGCGCTGGGACA	Δ*pelD* downstream 5'
vl494	5'-AAAGCTTTGTGTTCGGTCATGTCCAGTATCT	Δ*pelD* downstream 3'
vl745	5'-AGTTAACCTGGAGCGCCTGGAGCT	Δ*pelE* upstream 5'
vl746	5'-AGGATCCAGAGGAGCATATGTCTAAACAGCCACTTGCTGATCAT	Δ*pelE* upstream 3'
vl747	5'-ACATATGCTCCTCTGGATCCTGGCGAGATACTGGACATGA	Δ*pelE* downstream 5'
vl748	5'-AAAGCTTAAGGTCTTCACGTCCTTGATC	Δ*pelE* downstream 3'
vl627	5'-AGTTAACTGCGAGCGGACTGACGGCAAGCAA	*pel* promoter 5'
vl629	5'-ACGCTAGCCTACGCGGCAAGGTCGATA	*pel* promoter 3'

Fig. S1. Operon organization of *pelA-G*.
Predicted function, cellular location and alignment to homologous operons. Abbreviations under the genes are: SS, signal sequence; OM, outer membrane; PP, periplasmic; TM, transmembrane helix; IM, inner membrane.

BLAST score for each *pel* gene to each homolgue is indicated below each gene.

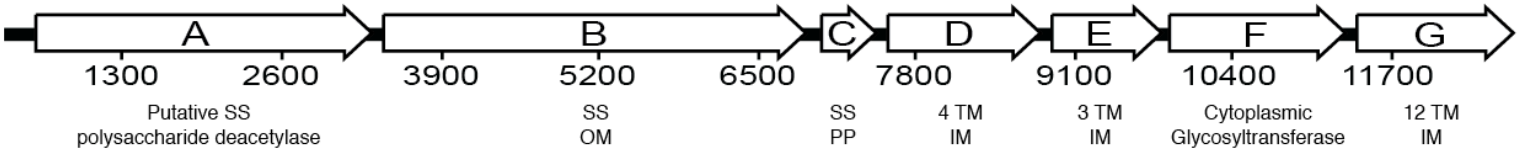

	A	B	C	D	E	F	G	PelD BLAST score
Pseudomonas aeruginosa	PA3064	PA3063	PA3062	PA3061	PA3060	PA3059	PA3058	
Pseudomonas fluorescens	PFL_2972	PFL_2973	PFL_2974	PFL_2975	PFL_2976	PFL_2977	PFL_2978	e-145
Marinobacter aqueolei	Maqu_2657	Maqu_2656	Maqu_2655	Maqu_2654	Maqu_2653	Maqu_2652		e-58
Pseudoalteromonas haloplanktis	PSHAa0709	PSHAa0708	PSHAa0707	PSHAa0706	PSHAa0705	PSHAa0704	PSHAa0703	e-54
Nitrosospira multiformis	Nmu1_A2432	Nmu1_A2441	Nmu1_A2437	Nmu1_A2436	Nmu1_A2435	Nmu1_A2434		e-54
Nitrosospira multiformis	Nmu1_A1736	Nmu1_A1735		Nmu1_A1734	Nmu1_A1733	Nmu1_A1732		e-48
Polaromonas naphthalenivorans	Pnap_0047	Pnap_0048		Pnap_0049		Pnap_0051		e-42
Ralstonia metallidurans	Rmet_4155	Rmet_4156	Rmet_4157	Rmet_4158	Rmet_4159	Rmet_4160		e-40
Geobacer metallireducens	Gmet1962	Gmet1964	Gmet1966	Gmet1967	Gmet1968	Gmet1969		e-40
Ralstonia solanacearum	RSc2276	RSc2275	RSc2274	RSc2273	RSc2272	RSc2271	RSc2270	e-37
Burkholderia cenocepacia	BcenP_6057	BcenP_6056	BcenP_6055	BcenP_6054		BcenP_6051		e-34
Burkholderia xenovorans	Bxe_A2975	Bxe_A2974	Bxe_A2973	Bxe_A2972	Bxe_A2971	Bxe_A2970		e-33

Supplemental Fig. 2. PelD does not bind cyclic-GMP or cyclic-AMP
The ability of either MBP or MBP-PelD to bind ^{3}H-c-di-GMP, ^{3}H-cyclic-GMP, or ^{3}H-cyclic-AMP was assessed by using the nucleotide binding assay as described. % bound of each above background is shown.

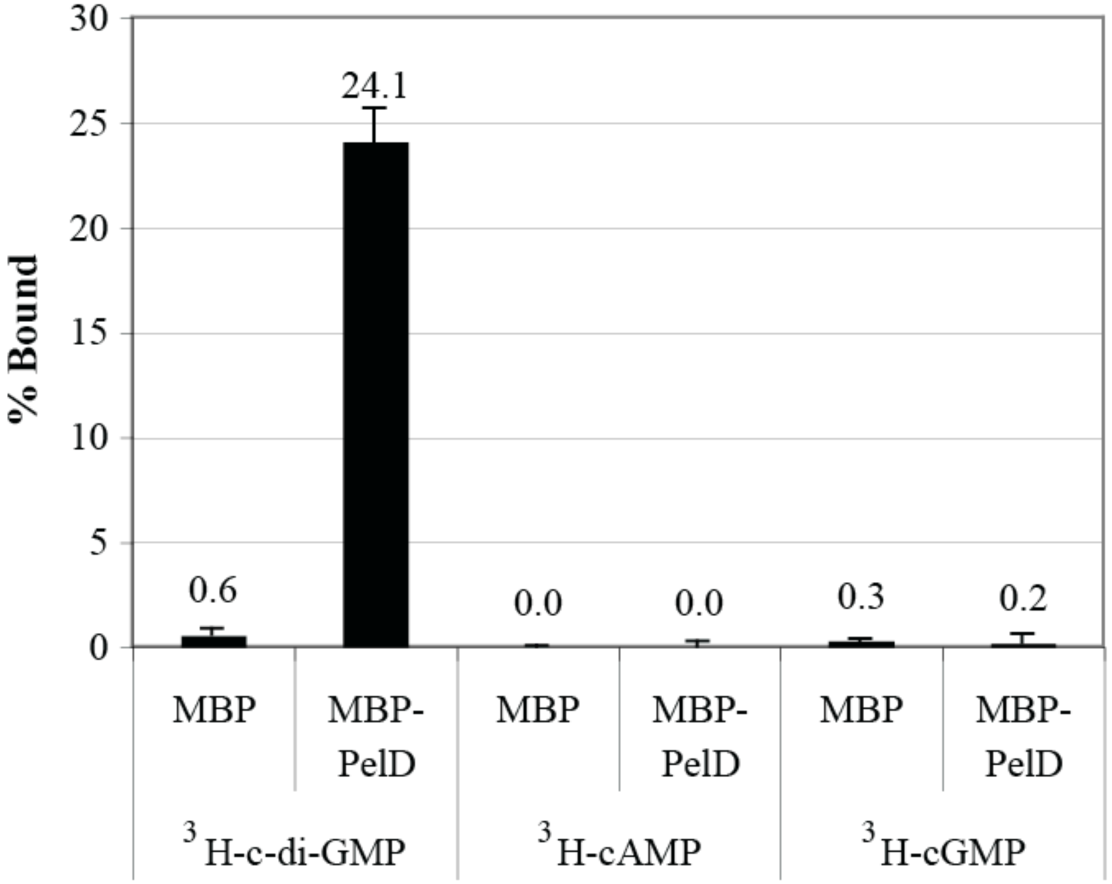

Fig. S3 Location of I-site and GGDEF motif on opposing faces of the PleD.

Location of RxxD (red), GGEEF (yellow) and other conserved residues (green) on PleD crystal structure. 1. Side view. 2. GGDEF face of PleD. 3. I-site face of PleD.

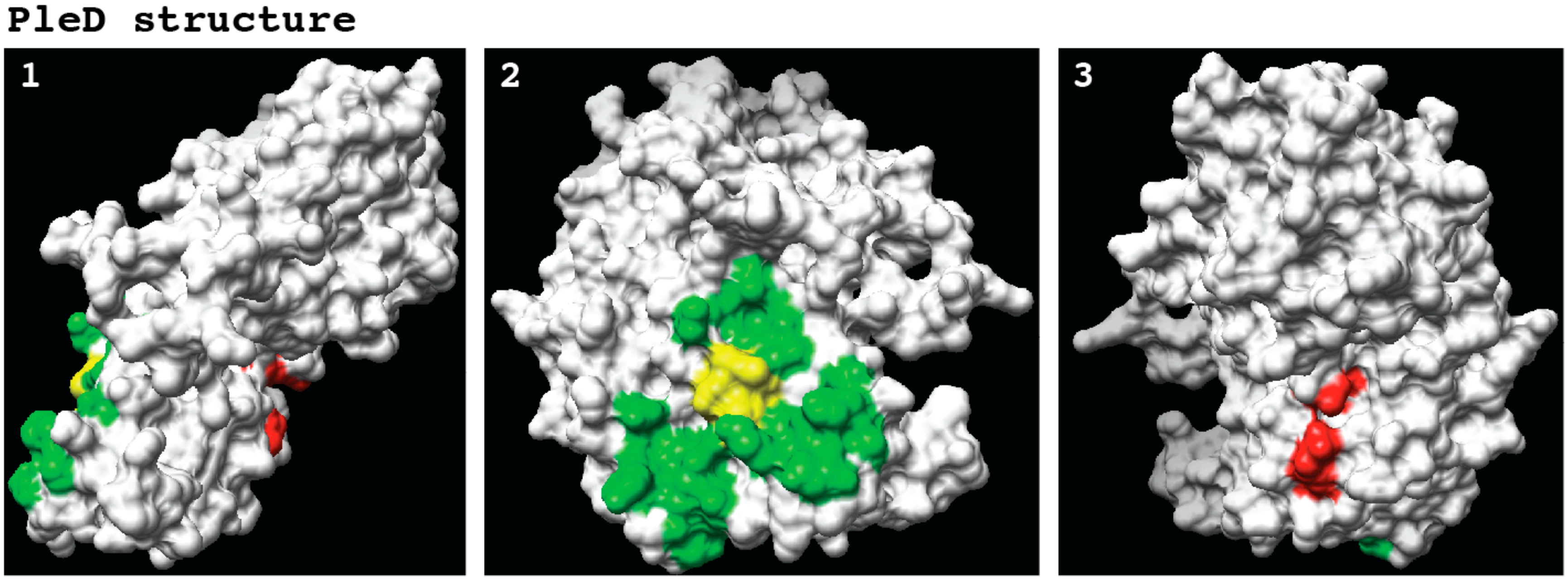

Supplement: Fig. S1 — Operon organization of pelA-G. Predicted function, cellular location and alignment to homologous operons. Abbreviations under the genes are: SS, signal sequence; OM, outer membrane; PP, periplasmic; TM, transmembrane helix; IM, inner membrane. BLAST score for each pel gene to each homologue is indicated below each gene. [file mmi0065-1474-SD1.pdf]
